# Efficacy, safety, and population pharmacokinetics of a single 1500mg dose of dalbavancin for short-term therapy in patients with chronic prosthetic joint infections

**DOI:** 10.1128/aac.00773-25

**Published:** 2025-10-17

**Authors:** Eva Benavent, Jaime Lora-Tamayo, Marta Ulldemolins, Paula Pons-Oltra, Matthieu Gregoire, Mikel Mancheño-Losa, Pilar Hernández-Jiménez, M. Ángeles Meléndez-Carmona, Victor Casals, Jason A. Roberts, Raul Rigo-Bonnin, Oscar Murillo

**Affiliations:** 1 Infectious Diseases Department, Hospital Universitari de Bellvitge, IDIBELL, Universitat de Barcelona, L’Hospitalet de Llobregat 16724, Barcelona, Spain; 2 Department of Internal Medicine, Hospital Universitario 12 de Octubre, Instituto de Investigación Imas12 16473, Madrid, Spain; 3 Centro de Investigación Biomédica en Red en Enfermedades Infecciosas (CIBERINFEC), Instituto de Salud Carlos III 38176, Madrid, Spain; 4 University of Queensland Centre for Clinical Research, Faculty of Medicine, The University of Queensland 1974, Brisbane, Australia; 5 Nantes Université, CHU Nantes, Service de Pharmacologie Clinique 27045, Nantes, France; 6 Nantes Université, CHU Nantes, Cibles et Médicaments des Infections et de l’Immunité, IICiMed, Nantes, France; 7 Department of Microbiology, Hospital Universitario 12 de Octubre, Instituto de Investigación Biomédica "imas12" Hospital 12 de Octubre, Madrid, Spain; 8 Orthopedic Surgery Department, Hospital Universitari de Bellvitge, IDIBELL, L’Hospitalet de Llobregat, Universitat de Barcelona 16724, Barcelona, Spain; 9 Herston Infectious Diseases Institute (HeIDI), Metro North Health, Brisbane, Australia; 10 Department of Pharmacy, Royal Brisbane and Women's Hospital 3883, Brisbane, Australia; 11 Department of Intensive Care Medicine, Royal Brisbane and Women's Hospital 550021, Brisbane, Australia; 12 Division of Anesthesia, Critical Care and Emergency and Pain Medicine, Nimes University Hospital, University of Montpellier, Nimes, France; 13 Department of Clinical Laboratory, Hospital Universitari de Bellvitge, IDIBELL, Universitat de Barcelona 16724, Barcelona, Spain; University of California San Francisco, San Francisco, California, USA

**Keywords:** long-acting antibiotic, two-stage prosthetic exchange, pharmacokinetic analysis, dalbavancin, chronic prosthetic joint infection

## Abstract

The efficacy, safety, and population pharmacokinetics of a single 1,500 mg dose of dalbavancin as a sequencing treatment for Gram-positive chronic prosthetic joint infections (CPJIs) have not been described. We present an observational, retrospective study conducted in two Spanish hospitals including patients with CPJI caused by Gram-positive bacteria susceptible to dalbavancin managed with two-stage exchange, antibiotic-loaded spacers, and a single 1,500 mg dose of dalbavancin. Follow-up visits included measurement of dalbavancin plasma concentrations. Negative intraoperative cultures at second-stage surgery defined microbiological cure. Population pharmacokinetics and Monte Carlo dosing simulations were used to evaluate whether this dose provided a therapeutic antibiotic exposure defined as the ratio between the area under the unbound concentration curve and the bacteria minimum inhibitory concentration (ƒAUC_0-24h_/MIC) ≥ 50 for the entire treatment period. Twenty patients were included, with CPJI mostly caused by coagulase-negative staphylococci (71%). After 11.5 days of intravenous antibiotic therapy (vancomycin, 75%), patients received 1,500 mg of dalbavancin without adverse events. Microbiological cure was 94.7% (median follow-up, 693 days). Dosing simulations suggest that a single 1,500 mg dose of dalbavancin is sufficient for maintaining ƒAUC_0-24h_/MIC ≥ 50 for MIC ≤ 0.25 mg/L for 3–4 weeks after administration. A single 1,500 mg dose of dalbavancin combined with antibiotic-loaded spacers may be an effective and safe sequencing treatment for CPJI and provide 3–4 weeks of therapeutic exposure for susceptible microorganisms. Considering dalbavancin’s unique pharmacokinetics, this approach may be considered in the clinical management of CPJI.

## INTRODUCTION

Chronic prosthetic joint infections (CPJIs) are often managed with two-stage revision surgery, exhibiting cure rates as high as 90%–95% (1–3). Initially, the infected prosthesis is removed, a broad-spectrum antibiotic-loaded cement spacer is implanted, and then systemic antibiotics are administered for 4–6 weeks with the aim of sterilizing the infection site for a new prosthesis to be implanted during the second-stage surgery (4, 5). Gram-positive bacteria, particularly coagulase-negative staphylococci (CoNS), are the most common causative agents for CPJI (6). They are often multidrug-resistant and may cause infections by multiple bacterial clones, for which the use of antibiotics with an extended spectrum against Gram-positive bacteria is beneficial in preventing persistence or superinfections at the time of prosthesis reimplantation (7).

Dalbavancin is a long-acting lipoglycopeptide antibiotic that provides prolonged antibiotic activity against many Gram-positive bacteria (8). Recent reports have documented off-label efficacy in bone and joint infections (9–11), including heterogeneous patient cohorts treated non-exclusively with single or repeated doses of dalbavancin. For CPJI managed with a two-stage exchange, dalbavancin is a sequencing option that may be advantageous in terms of efficacy, treatment adherence, and early hospital discharge, reducing the risks associated with prolonged hospitalizations and long parenteral or oral antibiotic courses.

Hence, we aimed to describe the efficacy and safety of a therapeutic strategy based on a single 1,500 mg dose of dalbavancin administered after first-stage exchange surgery in patients with CPJI. We also sought to describe the population pharmacokinetics (PK) of this single dose of dalbavancin to evaluate whether this dose provides a therapeutic antibiotic exposure for the entire treatment period in CPJI using Monte Carlo dosing simulations based on the final PK model.

## MATERIALS AND METHODS

### Study population and settings

We conducted a retrospective, observational, multicenter clinical and PK study that included adult patients who underwent two-stage prosthetic replacement for CPJI caused by low-virulent Gram-positive bacteria susceptible to dalbavancin and were treated with a single 1,500 mg dose of dalbavancin. The study was performed at specialized multidisciplinary units for managing complex osteoarticular infections from the Hospital Universitari de Bellvitge (Barcelona) and Hospital Universitario 12 Octubre (Madrid) from 1 January 2022 to 31 May 2023. This study received approval from the hospital’s ethics committee (reference EOM017/23).

### Clinical and surgical management

CPJI was defined according to established guidelines and international consensus (12, 13). All cases presented compatible clinical signs (pain, sinus tract), radiological prosthesis loosening, and intraoperative findings of purulent fluid and/or phenotypically indistinguishable microorganisms with identical antibiotic susceptibility pattern in at least two different samples from surgery. In all cases, patients underwent two-stage prosthetic joint replacement, with removal of the infected implant plus tissue debridement during the first-stage surgery as usually performed in routine practice. During surgery, several microbiological samples were collected from the surgical bed *as per* clinical practice, and then an antibiotic-loaded cement spacer containing vancomycin and gentamicin was implanted. All specimens underwent microbiological processing, with cultures incubated for 10 days under aerobic and anaerobic conditions. Microorganism identification and susceptibility testing followed the European Committee on Antimicrobial Susceptibility Testing (EUCAST) guidelines (14); concisely, vancomycin susceptibility was used as a surrogate to dalbavancin susceptibility (15, 16). After surgery, patients received empirical combined antibiotic therapy (i.e., vancomycin or daptomycin plus ceftazidime or cefepime) until Gram-positive etiology was confirmed, after which treatment was simplified to the anti-Gram-positive agent. After the post-operative period (typically around the first 2 weeks), the antibiotic treatment was sequenced to a single intravenous 1,500 mg dose of dalbavancin, given before hospital discharge over a 30-min short infusion (Hospital 12 de Octubre) or as a 2-h extended infusion (Hospital Universitari de Bellvitge) as per clinical practice of each institution. The post-operative treatment consisted of 2 weeks of standard intravenous antibiotics followed by a single dose of dalbavancin, which was expected to provide an additional 2 weeks of antibiotic treatment. This approach was intended to complete the standard 4 weeks treatment recommended after first-stage surgery for chronic prosthetic joint infections (4, 5).

### Follow-up and outcomes

Patients were followed weekly or biweekly in the outpatient clinic depending on clinical needs; during these visits, the adverse events attributable to dalbavancin were collected. As per usual clinical practice, analytical controls were performed in the follow-up visits and included the serial determination of dalbavancin total plasma concentrations until the fourth week post-administration. Infection cure was defined by (i) microbiological cure, defined as negative intraoperative cultures at second-stage surgery, and (ii) clinical cure, defined as the absence of clinical, radiological, and biological signs of infection with the same microorganisms that originally caused the infection.

### Sample handling and bioanalysis

Blood samples obtained during the follow-up visits were processed immediately after extraction, centrifuged at 3,000 g for 10 min and frozen at −80°C until bioanalysis. Total dalbavancin concentrations in plasma were measured using a previously developed and validated ultra-high-performance liquid chromatography–tandem mass spectrometry method (17). Lower limit of quantification, measuring interval, imprecisions, absolute relative bias, normalized-matrix factors, and normalized recoveries were 1.0 mg/L, 1.0–250 mg/L, ≤8.6%, ≤7.3 %, 96.4%–102.3%, and 95.1%–105.2% respectively. No carry-over or interferences were observed.

### Data collection and statistical analysis

Clinical, demographic, and PK data were anonymized and retrospectively collected in a database specifically designed for the study. The statistical analysis was performed with Stata 14.2 (Stata Corporation, Texas, USA). Categorical variables were described by counts and percentages, while medians and interquartile intervals (IQIs) were used to summarize continuous variables.

### Population pharmacokinetic analysis

The non-linear mixed-effect modeling software Monolix 2024R1 (Lixoft SAS, a Simulations Plus company) implementing the stochastic approximation expectation maximization algorithm was used for model development (18).

### Structural model building, covariate analysis, and model evaluation

One- and multicompartment models with first-order elimination were tested. All individual parameters were assumed to be log-normally distributed. Inter-individual variability (IIV) was described using an exponential model. To describe residual variability, several error models (constant, proportional, or combined error model) were tested. The most appropriate structural model was selected based on the following criteria: Akaike information criterion, corrected Bayesian information criterion, usual goodness-of-fit plots, and relative standard errors. Afterward, the effects of the following covariates on dalbavancin PK parameters were evaluated on the structural model: age, gender, height, body weight, body mass index, glomerular filtration rate (19), baseline creatinine clearance (CrCL) (20), and albumin serum concentrations on the day of sampling. The covariate model was built using a forward inclusion–backward elimination stepwise procedure, where covariates were kept in the model if they were plausibly related to the PK parameter, improved the fit, reduced between-subject variability, and significantly decreased the objective function value. Model internal validation was based on goodness-of-fit plots, including observations versus individual (IPRED) and population predictions (PRED) and plots of normalized prediction distribution error (NPDE) versus PRED and time. A prediction-corrected visual predictive check (pc-VPC) was performed using 500 simulations with the final model (21), and non-parametric bootstrapping (*n* = 1,000) was used for assessing stability and robustness of the final model using Monolix 2024R1.

### Monte Carlo dosing simulations

Monte Carlo simulations were performed using Simulx 2024R1 (Lixoft SAS, a Simulations Plus company) based on the final model for total dalbavancin. One thousand dalbavancin unbound concentrations over time profiles were generated for a single 1,500 mg dose administered over 30 min. Four different theoretical protein binding values were used for a 93%–99% protein binding range, accounting for the lowest described protein binding in the product information (93%) and recent data reporting 99% protein binding (22–24). Given that the pharmacokinetic/pharmacodynamic (PK/PD) target associated with efficacy for dalbavancin remains controversial, we chose a ratio of the area under the unbound concentration curve (ƒAUC_0-24h_) to the minimum inhibitory concentration (MIC) of the bacteria (ƒAUC_0-24h_/MIC) ≥ 50, as this target is associated with a 1-log_10_ kill from pre-clinical studies with *Staphylococcus aureus* (25, 26). This endpoint considers the recommendations of the European Medicines Agency and the Food and Drug Administration for intravenous antimicrobials and is also individualized to our patient population, who have deep-seated infections managed with a strong source-control strategy, i.e., two-stage implant exchange, extensive surgical debridement, and local antibiotic delivery via antibiotic-impregnated spacers, interventions that significantly reduce the bacterial inoculum (24, 27, 28). Furthermore, a stasis endpoint of (ƒAUC_0-24h_/MIC) ≥ 25 was also tested, accounting for a potential suppressive therapy indication. Since one of the aims of the study was to assess whether a 1,500 mg single dose of dalbavancin provides an adequate antibiotic exposure for the entire treatment period (4–6 weeks post-surgery), we evaluated the probability of target attainment (PTA) of this dose at different time points (3, 4, and 5 weeks post-dose) for a range of MIC from 0.002 to 0.25 mg/L. This range encompasses the most likely scenario (MIC 0.002–0.125 mg/L) and the worst-case scenario (MIC = 0.25 mg/L) for infections caused by susceptible Gram-positive bacteria, primarily *Staphylococcus* spp., according to the EUCAST epidemiological cut-off value (ECOFF) and clinical breakpoint respectively (14, 29). Optimal PTA was defined as ≥90% for all the analyzes.

## RESULTS

Twenty patients were included in the study, 70% of whom were female (n = 14). The median age was 75.5 years (IQI: 69–79), and all patients but two presented with comorbidities, the most frequent being systemic arterial hypertension (*n* = 5), dyslipidemia (*n* = 4), diabetes mellitus (*n* = 3), hypothyroidism (n = 3), and previous malignancy (n = 3). All patients had preserved renal function (median glomerular filtration rate 90 mL/min, IQI 75.8-96.3 mL/min) and normal albumin serum concentrations (median 45 g/L, IQI 38–47 g/L). Infection involved hip prosthesis in most patients (55%; *n* = 11), followed by knee prosthesis (30%; *n* = 6), shoulder prosthesis (10%; *n* = 2), and one case of ankle prosthesis (5%). The isolates responsible for CPJI are presented in Table 1; there were four cases of polymicrobial infection.

**TABLE 1:** Isolated microorganisms in the cultures from the first-stage surgery

Microbiological isolates	*n* = 24	(%)^*a*^
Coagulase-negative staphylococci (CoNS)	17	70.8%
*Staphylococcus epidermidis*	12	50%
*Staphylococcus lugdunensis*	1	4.2%
Other CoNS^*b*^	4	16.7%
*Cutibacterium acnes*	6	25%
*Enterococcus faecalis*	1	4.2%
Polymicrobial infections^*c*^	4	20%

^*a*^ From 20 episodes.

^*b*^ 2 *Staphylococcus capiti*s, 1 *Staphylococcus hominis,* and 1 *Staphylococcus pettenkoferi*. MIC: minimum inhibitory concentration.

^*c*^ *Cutibacterium acnes* was involved in 4 polymicrobial infections, in all cases coexisting with coagulase-negative staphylococci.

**Fig 1:**
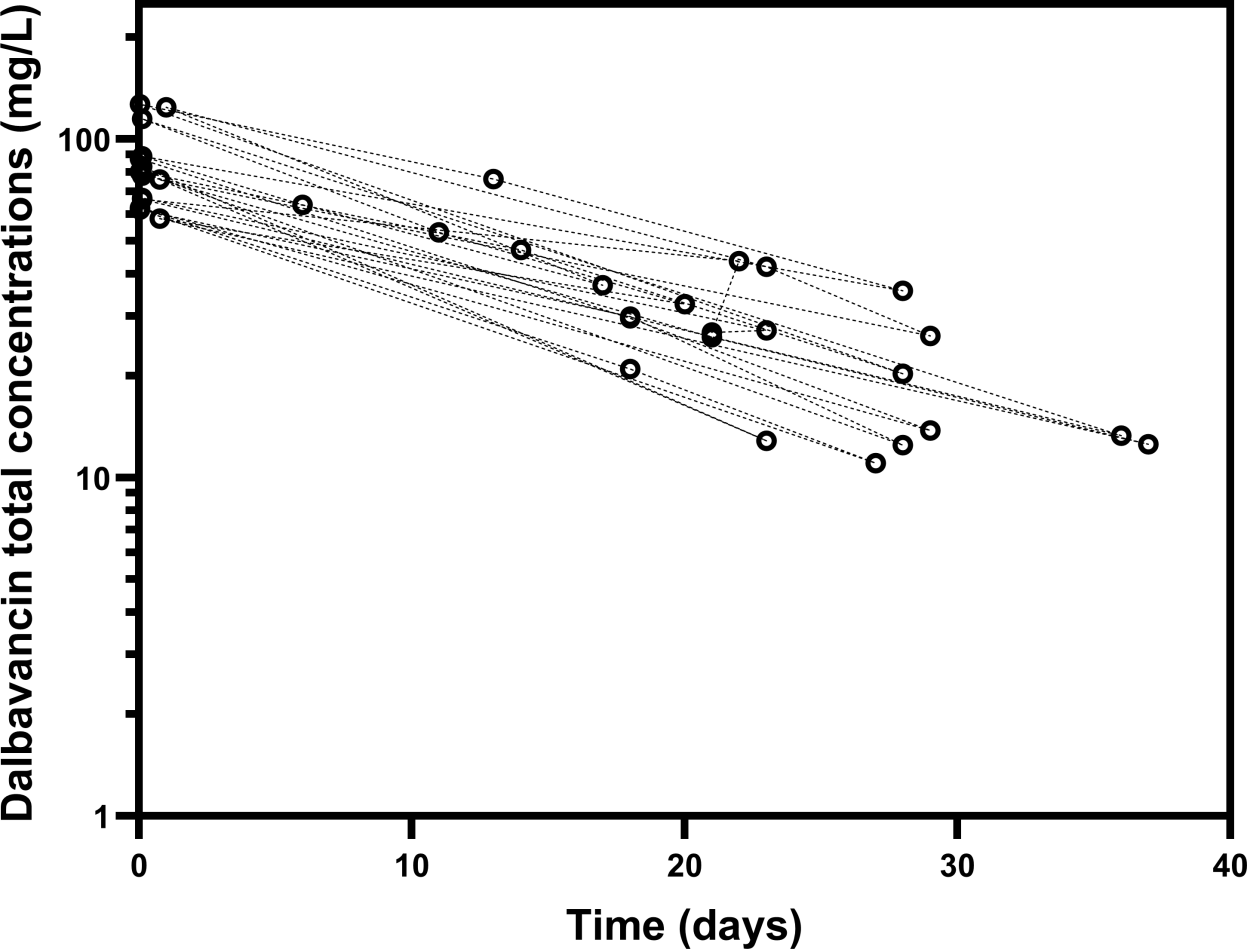
Observed total concentrations over time of dalbavancin during the follow-up period. Concentrations are in mg/L, time is in days. The Y-axis is displayed using a semi-logarithmic scale.

After a median of 11.5 days of systemic antibiotic treatment (IQI: 10–16 days), patients received a single 1,500 mg dose of dalbavancin in monotherapy. Initially, 15 cases received vancomycin and 5 daptomycin, and in 30% of the cases, these intravenous antibiotics were switched to oxazolidinones prior to the administration of dalbavancin (Table S1). Figure 1 represents the total concentrations over time of dalbavancin over the follow-up period. Overall, dalbavancin median trough concentration was 58.5 mg/L (IQI 50–70) after 2 weeks post-administration (*n* = 4; day 12 [IQI 8.5–13.5]); 29.7 mg/L (IQI 26.8–37.1) after 3 weeks (*n* = 10; day 20.5 [IQI 18–22]) and 13.3 mg/L (IQI 12.55–20.3) after 4 weeks (*n* = 9; day 28 [IQI 28–29]). No adverse events were reported over administration and during the follow-up, including changes in renal function.

### Follow-up and outcome

All but one patient underwent second-stage surgery after a median time of 134 days (IQI 85–175) from the first surgery. The microbiological cure rate was 94.7%, i.e., all patients except for one that had an initial infection caused by *Cutibacterium acnes*, but *Staphylococcus epidermidis* and *Staphylococcus hominis* grew in the intraoperative cultures from the second-stage surgery. Although those isolates were susceptible to dalbavancin, the patient received tedizolid for six extra weeks. Only one patient declined second-stage surgery because she had good functionality with the spacer and was considered clinically cured. There were no clinical failures or relapses after a median follow-up of 693 days (IQI 630–824) after the second-stage procedure.

### Population pharmacokinetic analysis and Monte Carlo dosing simulations

We developed a population PK model with total dalbavancin concentration data from 18 patients (Table S2); each patient contributed with 1–3 concentrations. The structural model that best described dalbavancin total concentrations over time was a one-compartment model with intravenous infusion and first-order linear elimination. Interindividual variability was included in both volume of distribution (V) and clearance (CL), and the residual error was modeled as proportional. The covariate analysis did not result in model improvements for which the structural model is the final model, summarized in Table 2. The internal validation showed model stability without model misspecifications (Table 2 and Fig. S1 and S2). The results of Monte Carlo simulations are summarized in Table 3 and Table S3 and suggest that a 1,500 mg single dose of dalbavancin provides therapeutic unbound exposure in plasma for the PK/PD target of 1-log_10_ kill and bacteriostasis (ƒAUC_0-24h_/MIC) ≥50 and ≥25 respectively, against microorganisms with MIC between 0.002 and 0.125 mg/L for at least 3 weeks post-administration, for all protein binding values tested. For a protein binding ≤97%, this dose would be sufficient for attaining therapeutic exposure in plasma for microorganisms with higher MIC (≤0.25 mg/L, corresponding to the clinical breakpoint for *Staphylococcus* spp.) for at least 4 weeks post-administration. In the most favorable protein binding scenario (93%), simulations suggest that therapeutic exposure in plasma is maintained for at least 5 weeks post-administration.

**TABLE 2:** Population pharmacokinetic estimates for dalbavancin^*a*^

Parameter	Estimate (% RSE) [Shrinkage %]	Bootstrap median (95% CI)
Fixed effects		
V (L)	17.9 (6.2 %) [18.1%]	18.0 (16.0–20.3)
CL (L/h)	0.036 (8.2 %) [12.6%]	0.037 (0.031–0.043)
Random effects		
IIV V (SD)	0.200 (27.4 %)	0.200 (0.061–0.280)
IIV CL (SD)	0.290 (20.8 %)	0.280 (0.150–0.380)
Residual variability		
b (proportional)	0.120 (28. 6%)	0.110 (0.053–0.170)

^*a*^ V, volume of distribution; CL, clearance; IIV V, interindividual variability on volume of distribution; IIV CL, interindividual variability on clearance; SD, standard deviation; RSE, relative standard error; CI, confidence interval.

**TABLE 3:** Probability of target attainment (PTA) of a 1,500 mg single dose of dalbavancin (PK/PD target of ƒAUC_0-24h_/MIC ≥ 50) considering typical MICs and a worst-case scenario (MIC = 0.25 mg/L) for infections caused by susceptible *Staphylococcus* spp. (14, 29)^*a*^

PK/PD target		ƒAUC_0-24h_/MIC ≥50			
MIC (mg/L)		≤ 0.030	0.060	0.125	0.250
Protein binding (%)	Days post-administration				
93%	Day 21 (end of week 3)	100	100	100	99.7
	Day 27 (end of week 4)	100	100	99.6	97.5
	Day 35 (end of week 5)	100	99.5	97.2	85
95%	Day 21 (end of week 3)	100	100	100	99.3
	Day 27 (end of week 4)	100	100	98.8	92.8
	Day 35 (end of week 5)	99.6	98.7	93.3	68.9
97%	Day 21 (end of week 3)	100	100	99.6	93.4
	Day 27 (end of week 4)	100	99.6	95.5	66.9
	Day 35 (end of week 5)	99.3	96.2	79	30.6
99%	Day 21 (end of week 3)	100	100	98.4	65.9
	Day 27 (end of week 4)	99.7	98.2	87.3	28.2
	Day 35 (end of week 5)	97.8	89.6	57	5.4

^*a*^ The results are stratified by theoretical protein binding. Dark gray-shaded areas correspond to a PTA rounded up to 405 ≥ 90%.

## DISCUSSION

To our knowledge, this is the first study that reports the efficacy, safety, population PK and dosing simulations of a 1,500 mg single dose of dalbavancin as a sequencing therapy for the treatment of CPJIs caused by Gram-positive bacteria managed with a two-stage exchange surgery. Our data show that the administration of a single 1,500 mg dose of dalbavancin after 1–2 weeks of prior antibiotic therapy during hospitalization and in combination with antibiotic-loaded spacers containing vancomycin plus gentamicin results in a 94.7% cure rate after long-term follow-up, with an excellent safety profile. The population PK analysis and dosing simulations are congruent with these outcomes, suggesting that this strategy provides therapeutic unbound exposure in plasma for 3–4 weeks post-administration, depending on the MIC and the protein binding. Overall, this strategy based on a single 1,500 mg dose of dalbavancin would complete an adequate treatment course for CPJI.

Dalbavancin is a long-acting lipoglycopeptide that has been used off-label in osteoarticular infections (2, 9, 11, 30–32). In the context of CPJIs managed with two-stage prosthetic replacement, it may be a valuable sequencing option that facilitates early discharge, ensures treatment compliance, and, due to its safety profile, minimize the risk of interactions and adverse effects compared with other systemic treatments like oxazolidinones. Our experience in a cohort of patients with CPJI caused by common Gram-positive microorganisms supports this approach, with a high clinical cure rate later confirmed, with sterile cultures during the second-stage surgery and long-term follow-up and no observed remarkable adverse events over the subsequent weeks, which aligns with previous findings (9, 33). Notably, favorable outcomes were observed in cases caused by *Cutibacterium acnes* and enterococci, despite the absence of EUCAST susceptibility breakpoints for dalbavancin against these organisms. While vancomycin susceptibility is often used as a surrogate for dalbavancin susceptibility (16), previous studies have also evaluated the *in vitro* activity and clinical efficacy of dalbavancin in infections caused by these microorganisms (16, 34–36). In this context, in addition to dalbavancin’s efficacy, the intravenous antibiotic administered during the initial post-operative period (i.e., vancomycin, daptomycin, or linezolid), along with the local antibiotic delivery via spacers, may also contribute significantly to microbiological cure. Finally, no remarkable adverse events were observed over the subsequent weeks post-administration of dalbavancin, which aligns with previous findings (9, 15), although our small sample size might have limited the ability to quantify them.

Assessment of optimal antibiotic exposure at the site of the infection is challenging, especially for deep-seated infections, such as bone and joint, and often unbound plasma exposure is used as a worst-case scenario surrogate for target site exposure assuming that only the free (unbound) concentration can distribute to peripheral tissues (37). However, drug PK in the peripheral tissues depends not only on protein binding but also on other characteristics, such as drug’s molecular weight and physiochemistry, varying broadly across antibiotic classes (38). For dalbavancin, Dunne et al. performed a PK study in plasma and cortical bone in non-infected patients undergoing elective orthopedic surgery and reported that a single 1,000 mg dose of dalbavancin resulted in a measured amount of 4.1 µg/g of homogenized cortical bone 2 weeks post-administration; this translated to an estimated 13.1% penetration ratio (AUC_bone_/AUC_plasma_) obtained by population PK modeling, which would be higher than the one expected from the unbound concentrations approach (39). However, this study was performed in patients with healthy bones, and drug penetration was estimated from the amount of drug measured in homogenized cortical bone from sparse sampling, for which extrapolation to our patient cohort is uncertain. Our Monte Carlo simulations were, therefore, based on the conservative approach, considering the unbound concentrations in plasma for calculating target attainment and showed that therapeutic dalbavancin exposure was attained for at least 3–4 weeks of treatment even for microorganisms with MIC close to the susceptibility breakpoint (Table 3). These results are supportive of the observed good clinical and microbiological outcomes in our cohort.

Notably, we report dalbavancin’s pharmacokinetics with a single 1,500 mg dose over a 4-week period in elderly patients with CPJI, a population not well-described previously. Our observations were best described by a one-compartment model, which is in accordance with the sparse data gathered from routine clinical practice (40). Interestingly, the model estimates for CL are 30% lower than the one reported in healthy volunteers and patients with acute infections (0.036 L/h vs 0.050 L/h, respectively) (22, 41). A potential explanation is that the studies that report higher CL were performed in healthy volunteers and younger patients with acute infections that presented with lower plasma albumin concentrations and/or higher CrCL, characteristics that had an impact on dalbavancin CL in the population PK models (22, 41); on the other hand, our patients were older, had physiological albumin concentrations, and lower CrCL. The lower drug CL in our cohort may also justify the higher total dalbavancin concentrations observed 4 weeks after dose administration, which also align with Hervochon *et al.*, who report similar drug concentrations over time in a cohort of elderly patients with diverse infections (42). These concentrations are also above the threshold of 8 mg/L recommended by expert consensus as the trough total dalbavancin concentration associated with therapeutic exposure against staphylococci (43), supporting as well the results of our PTA analysis that suggests therapeutic exposure at this time point. Being our study specific for patients with CPJI, our results suggest that dalbavancin 1,500 mg doses could be spaced up to 4 weeks if repeated infusions were required in this population, for example, for suppressive therapy. However, further studies validating our data in this clinical setting are required.

Our study acknowledges several limitations. The small sample size may limit the detection of additional failures or adverse events, but the confirmed microbiological cure rate at the time of second-stage surgery and the prolonged patient follow-up (almost 2 years) reduce the risk of non-identified late failures. Also, the possibility that persistent dalbavancin bone concentrations were responsible for inhibiting the microorganisms’ growth in the intraoperative cultures rather than achieving true sterilization at the time of second-stage surgery is reasonably precluded by the fact that the surgery was performed after a median of 140 days (IQR: 85––175) after dalbavancin administration. Additionally, measurement of plasma concentrations was guided by clinical follow-up needs rather than a systematic approach, in line with standard clinical practice. Finally, our cases represent the most common causative agents of CPJI, which should not be generalized to other microorganisms; furthermore, our cohort presented normal renal function and serum albumin concentrations, which may limit the generalizability of our findings to patients with different clinical conditions. Despite these limitations, this real-life study, focused on CPJI caused by Gram-positive bacteria, benefits from the expertise of two specialized multidisciplinary units in complex osteoarticular infections and is accompanied by PK data that support our clinical findings.

In conclusion, our proposed simplified antibiotic regimen centered around a single 1,500 mg dose of dalbavancin together with antibiotic-loaded spacers offers a pragmatic and effective approach to manage CPJI by optimizing exposure, ensuring compliance, and minimizing toxicity. Further, our results suggest that dalbavancin 1500 mg doses could be spaced up to 4 weeks if repeated doses were required in this patient population, findings that need to be confirmed with additional research. Leveraging dalbavancin’s unique PK, this approach could potentially streamline treatment protocols and improve patient-centered outcomes.

## SUPPLEMENTAL MATERIAL

Supplemental material aac.00773-25-s0001.docx Tables S1 to S3; Fig. S1 and S2.

ASM does not own the copyrights to Supplemental Material that may be linked to, or accessed through, an article. The authors have granted ASM a non-exclusive, world-wide license to publish the Supplemental Material files. Please contact the corresponding author directly for reuse.
