# Supplementary material for: Efficacy, safety, and population pharmacokinetics of a single 1500mg dose of dalbavancin for short-term therapy in patients with chronic prosthetic joint infections
Source: Antimicrob Agents Chemother. 2025 Oct 17;69(12):e00773-25. doi: 10.1128/aac.00773-25 (PMC12691635; doi:10.1128/aac.00773-25)
Supplement: Supplemental material — Tables S1 to S3; Fig. S1 and S2. [file aac.00773-25-s0001.docx]

**SUPPLEMENTARY MATERIAL FOR RESULTS**

**TABLE S1:** Chronological overview of patients’ management and clinical course

| **Patient**  **Prosthesis** | **First-stage surgery** | | | **Second-stage surgery** | |
| --- | --- | --- | --- | --- | --- |
|  | **Date** | **Isolates** | **Antibiotics timing (days)** | **Date** | **Isolates** |
| 1  Ankle | November 2022 | *S. epidermidis* | Daptomycin (4)-Linezolid (10)  Dalbavancin (single dose) | Not performed |  |
| 2  Hip | November 2022 | *S. lugdunensis* | Daptomycin (4)-Cefazolin (7)  Dalbavancin (single dose) | February 2023 | No growth |
| 3  Hip | December 2022 | *E. faecalis* | Daptomycin (4)-Ampicillin (7)  Dalbavancin (single dose) | January 2023 | No growth |
| 4  Hip | December 2022 | *S. hominis* | Vancomycin (6)-Linezolid (9)  Dalbavancin (single dose) | March 2023 | No growth |
| 5  Hip | May 2023 | *S. epidermidis* | Daptomycin (10)  Dalbavancin (single dose) | September 2023 | No growth |
| 6  Hip | January 2023 | *S. epidermidis* | Vancomycin (13)-Daptomycin (3)  Dalbavancin (single dose) | June 2023 | No growth |
| 7  Shoulder | May 2023 | *S. epidermidis* | Vancomycin (3)-Daptomycin (5)  Dalbavancin (single dose) | September 2023 | No growth |
| 8  Knee | May 2023 | *C. acnes* | Vancomycin (8)  Dalbavancin (single dose) | September 2023 | No growth |
| 9  Hip | January 2023 | *S. epidermidis* | Vancomycin (20)  Dalbavancin (single dose) | July 2023 | No growth |
| 10  Hip | July 2022 | *S. epidermidis*  *C. acnes* | Vancomycin (3)-Daptomycin (9)  Dalbavancin (single dose) | December 2022 | No growth |
| 11  Knee | October 2022 | *S. epidermidis* | Vancomycin (7)  Dalbavancin (single dose) | March 2023 | No growth |
| 12  Knee | May 2023 | *C. acnes* | Vancomycin (3)-Linezolid (5)  Dalbavancin (single dose) | September 2023 | No growth |
| 13  Knee | September 2022 | *S. epidermidis* | Vancomycin (10)  Dalbavancin (single dose) | April 2023 | No growth |
| 14  Knee | February 2023 | *S. epidermidis* | Vancomycin (7)-Linezolid (5)  Dalbavancin (single dose) | June 2023 | No growth |
| 15  Hip | March 2023 | *S. capitis*  *C. acnes* | Vancomycin (7)-Daptomycin (10)  Dalbavancin (single dose) | July 2023 | No growth |
| 16  Knee | May 2023 | *S. capitis*  *S. pettenkoferi* | Vancomycin (5)-Daptomycin (7)  Dalbavancin (single dose) | September 2023 | No growth |
| 17  Shoulder | July 2022 | *C. acnes* | Vancomycin (10)  Dalbavancin (single dose) | October 2022 | *S. epidermidis*  *S. hominis* |
| 18  Hip | August 2022 | *S. epidermidis* | Vancomycin (10)-Linezolid (13)  Dalbavancin (single dose) | February 2023 | No growth |
| 19  Hip | May 2023 | *S. epidermidis*  *C. acnes* | Vancomycin (4)-Tedizolid (14)  Dalbavancin (single dose) | September 2023 | No growth |
| 20  Hip | December 2022 | *S. epidermidis* | Daptomycin (12)  Dalbavancin (single dose) | March 2023 | No growth |

**TABLE S2.** Baseline demographical and clinical characteristics of the 18 patients whose data was used for the population PK model development. Data are described as median (interquartile interval, quartile 1 – quartile 3) or count (percentage, %) as appropriated.

| **Variable** | n = 18 |
| --- | --- |
| Age (years) | 77 (69 – 79) |
| Sex (Females) | 11 (61.1 %) |
| Height (cm) | 165 (158 – 169) |
| Weight (kg) | 79 (73.1 – 82.1) |
| Body mass index (kg/m2) | 28.4 (26.8 – 31.6) |
| Hospital of admission (HUB/H12O) | 13 (72.5 %) / 5 (27.8 %) |
| Affected joint |  |
| Hip | 10 (55.6 %) |
| Knee | 5 (27.8 %) |
| Shoulder | 2 (11.1 %) |
| Ankle | 1 (5.6 %) |
| Creatinine concentration at admission (mg/dL) | 0.6 (0.6 – 1.1) |
| eGFR (mL/min) (1) | 90 (75.8 - 96.3) |
| CrCL (mL/min) (2) | 83.5 (68.5 – 92.8) |
| Serum albumin concentration (g/L) | 45 (38 – 47) |

eGFR: estimated glomerular filtration rate, CrCL: Creatinine clearance, HUB: Hospital Universitari de Bellvitge, H12O: Hospital Universitario 12 de Octubre.

**TABLE S3**: Probability of target attainment (PTA) of a 1500mg single dose of dalbavancin (PK/PD target of ƒAUC_0-24h_/MIC ≥ 25, stasis breakpoint) considering typical MICs and a worst-case scenario (MIC = 0.25 mg/L) for infections caused by susceptible *Staphylococcus* spp (3, 4).

| **MIC (mg/L)** | | **≤ 0.030** | **0.060** | **0.125** | **0.250** |
| --- | --- | --- | --- | --- | --- |
| **Protein binding (%)** | **Days post-administration** |  | | | |
| **93 %** | **Day 21**  **(end of week 3)** | 100 | 100 | 100 | 100 |
|  | **Day 27**  **(end of week 4)** | 100 | 100 | 100 | 99.6 |
|  | **Day 35**  **(end of week 5)** | 100 | 99.8 | 99.5 | 97.2 |
| **95 %** | **Day 21**  **(end of week 3)** | 100 | 100 | 100 | 100 |
|  | **Day 27**  **(end of week 4)** | 100 | 100 | 99.8 | 98.8 |
|  | **Day 35**  **(end of week 5)** | 100 | 99.6 | 98.5 | 93.3 |
| **97 %** | **Day 21**  **(end of week 3)** | 100 | 100 | 100 | 99.6 |
|  | **Day 27**  **(end of week 4)** | 100 | 100 | 99.6 | 95.5 |
|  | **Day 35**  **(end of week 5)** | 99.7 | 99.3 | 95.7 | 79 |
| **99 %** | **Day 21**  **(end of week 3)** | 100 | 100 | 98.2 | 65.9 |
|  | **Day 27**  **(end of week 4)** | 99.7 | 98.2 | 85.9 | 28.2 |
|  | **Day 35**  **(end of week 5)** | 97.8 | 89.6 | 53.8 | 5.4 |

The results are stratified by theoretical protein binding. Dark grey shaded areas correspond to a PTA rounded up to ≥ 90 %.

### **FIGURE S1:** Goodness-of-fit plots for the final population pharmacokinetic model of dalbavancin. Upper panels: (left) observed total concentrations versus population predicted concentrations and (right) observed total concentrations versus individual predicted concentrations. Lower panels: (left) normalised prediction distribution errors (NPDE) versus time and (right) NPDE versus population predicted concentrations. Blue dots represent the observations. Black solid lines represent line of unity. Red dashed lines represent spline lines.

### **
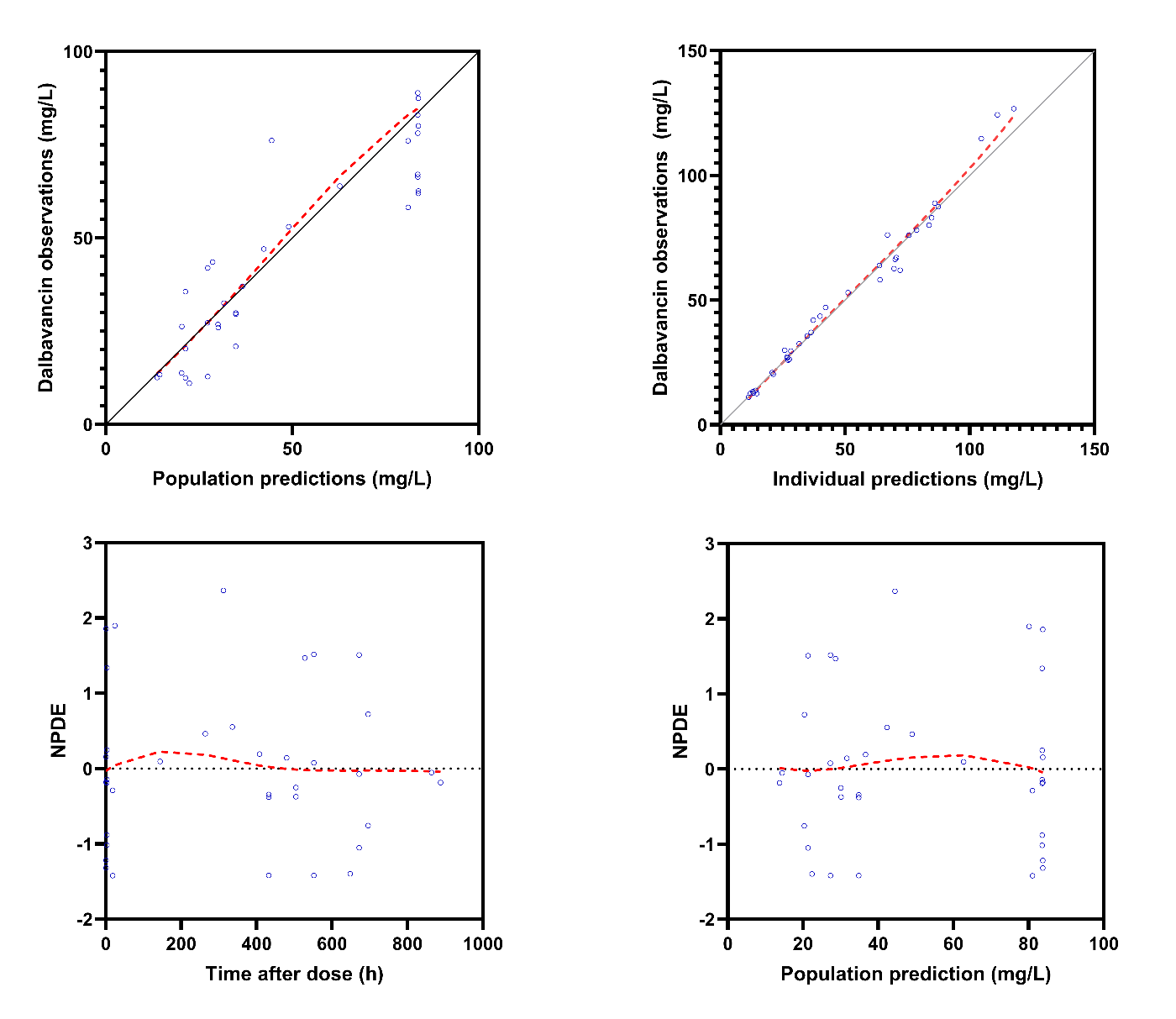
**

### **FIGURE S2:** Prediction-corrected visual predictive check for dalbavancin total plasma concentrations. The solid black line represents the 50^th^ empirical percentile of the observed data, and the dashed black lines represent the 10^th^ and 90^th^ empirical percentiles of observed data. The pink shaded area represents the prediction interval of the 50^th^ percentile, and the blue areas represent the 90% prediction intervals of the 10^th^ and 90^th^ percentiles. The empty blue dots represent the observed data.


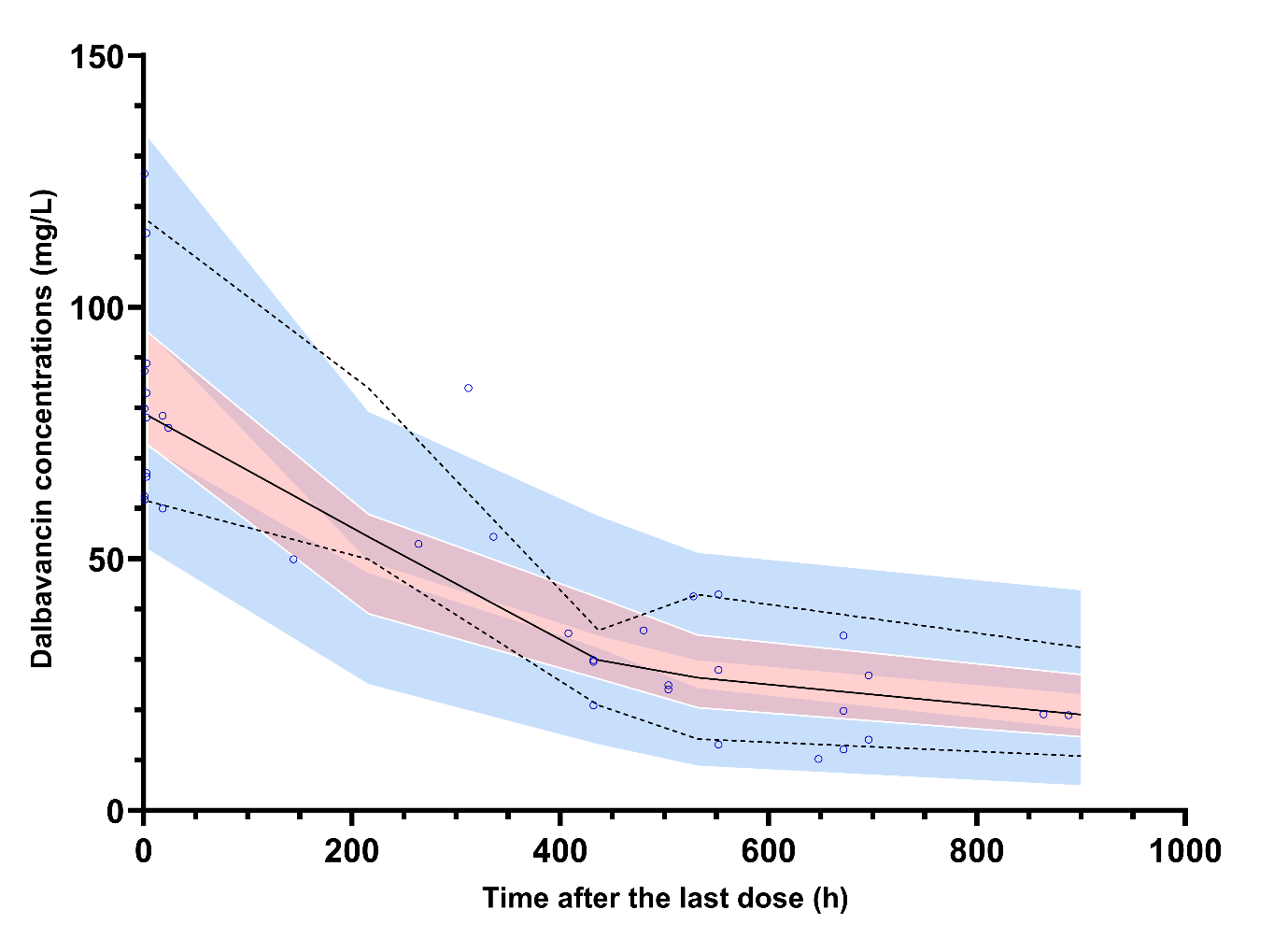


**REFERENCES FOR THE SUPPLEMENTARY MATERIAL**

1. Levey AS, Stevens LA, Schmid CH, Zhang YL, Castro AF, 3rd, Feldman HI, Kusek JW, Eggers P, Van Lente F, Greene T, Coresh J, Ckd EPI. 2009. A new equation to estimate glomerular filtration rate. Ann Intern Med 150:604-12.

2. Cockcroft DW, Gault MH. 1976. Prediction of creatinine clearance from serum creatinine. Nephron 16:31-41.

3. European Committee on Antimicrobial Susceptibility Testing (EUCAST). MIC and zone diameter distributions and ECOFFs. Accessible from wwweucastorg.

4. European Committee on Antimicrobial Susceptibility Testing (EUCAST). Clinical breakpoints and dosing. Accessible from: https://www.eucast.org/clinical_breakpoints.
